# It Keeps Getting Bigger: A Patient With a Hypothalamic Tumor and Hyperprolactinemia

**DOI:** 10.7759/cureus.80793

**Published:** 2025-03-18

**Authors:** Musa Kiyani, Marvin Wei Jie Chua

**Affiliations:** 1 General Medicine, Sengkang General Hospital, Singapore, SGP

**Keywords:** brain tumors, end stage renal disease (esrd), hyperprolactinemia, hypothalamic tumor, prolactin

## Abstract

Internists might occasionally see patients with prolactinomas and non-prolactin-secreting intracranial tumors under their care. This report presents an interesting and rare case of a woman with end-stage renal disease, who presented with hallucinations and a suprasellar mass. Her symptoms and prolactin levels, however, did not improve with adherent, inpatient, dopamine agonist treatment. She was eventually palliated and demised shortly after compassionate discharge. It is imperative that physicians maintain a high index of suspicion of alternative diagnoses (such as metastatic disease or high-grade glioma) when hypothalamic masses and raised prolactin levels do not significantly decline with dopamine agonism.

## Introduction

Prolactinomas and non-prolactin-secreting masses are rare but not uncommon. They account for approximately 40% of all pituitary neuroendocrine tumors, with a preponderance in women aged 20-50 years [1]. Mass effects (e.g. bitemporal hemianopia and headaches) can occur with macroprolactinomas, while infertility and hypogonadism may present with both small and large pituitary tumors [2]. Treatment, either medical or surgical, is intended to control excess hormone production and reduce mass effects.

Although prolactin levels generally parallel tumor size and patients with prolactin concentrations greater than 250 µg/liter do often have a prolactinoma, this is not always true. Hyperprolactinemia might be a red herring, especially in instances of renal disease, hypothyroidism, and hypothalamic metastatic diseases [3]. Furthermore, certain features are suggestive of more aggressive pituitary tumors, including corticotroph and somatotroph invasive macroadenomas, and those that undergo rapid progression despite radiotherapy or surgical removal [4].

Here, we discuss an interesting case of a patient with end-stage renal disease (ESRD) who presented with a new, symptomatic, hypothalamic mass.

## Case presentation

The patient, a middle-aged lady, was initially brought to our hospital in late April 2024 for episodes of visual hallucination and confusion in the preceding week. There were no other symptoms of psychosis such as auditory hallucinations, delusions, or behavioral agitation. There were no infective or neurological symptoms and no change in prescribed or alternative medications. According to her family, there was no recent trauma or head injury. She had a known history of ESRD secondary to diabetic kidney disease, renal cell carcinoma (T3, grade 3) status-post right, open, radical nephrectomy 10 years prior to presentation, heart failure with preserved ejection fraction, pulmonary hypertension, and obstructive sleep apnea.

**Figure 1:**
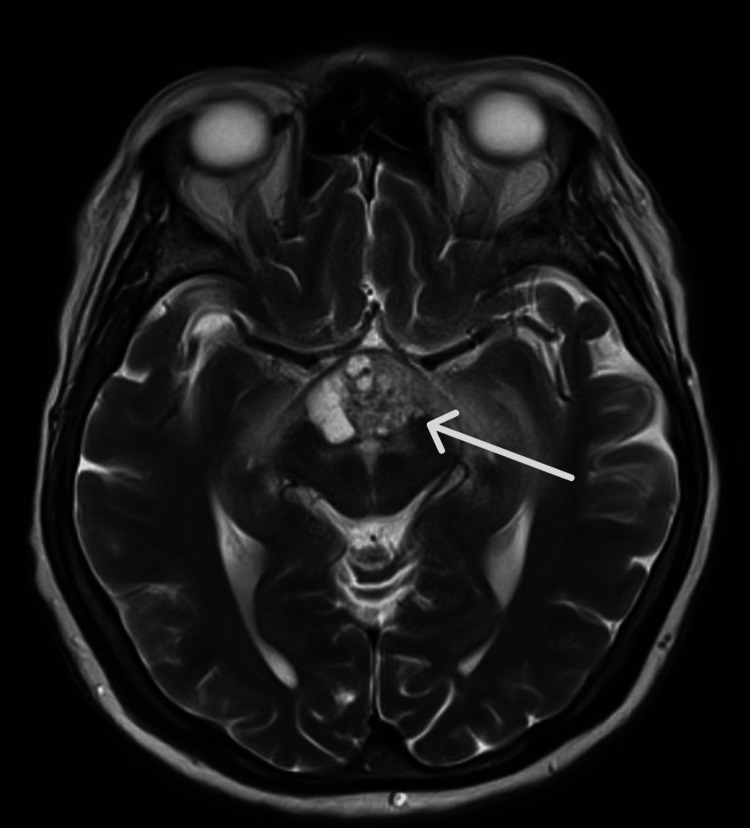
Non-contrast MRI brain with (axial T2) MRA performed on April 26, 2024 Relevant findings include a midline suprasellar hypothalamic heterogeneous solid-cystic mass, with local mass effect and perilesional edema, as well as apparent extension to the optic chiasm, but no overt sellar involvement. The mass measures up to 30x20 mm in the axial plane and 17 mm in the craniocaudal direction. Susceptibility suggesting hemorrhage is seen in the solid component. The third ventricle floor is indented superiorly. There is edema along the optic radiations, as well as mass effect and edema involving the midbrain and thalami bilaterally. MRA: magnetic resonance angiography

On initial assessment, the Glasgow Coma Scale (GCS) score was 14 with no focal neurological deficits, and the patient was assessed to be in acute delirium, as evidenced by fluctuating mentation and inattention. The tunneled dialysis catheter site was clean, with no warmth or fluctuance. The rest of the physical examination was unremarkable. Her infectious and metabolic screens were unchanged from prior admissions. An initial computed tomography (CT) brain had findings equivocal for an infarct, following which magnetic resonance imaging (MRI) brain was performed for further evaluation. Here, things started to unravel: MRI (Figure 1) demonstrated a new suprasellar/hypothalamic solid-cystic lesion. Hormonal screening (Table 1) was consistent with secondary adrenal insufficiency and hypothyroidism, as well as markedly elevated prolactin levels, notwithstanding the fact that the patient had ESRD which could contribute to reduced prolactin degradation and clearance, increased production, and altered regulation [5]. 

**Table 1:** Hormonal concentrations in the plasma/serum at different times Bolded numbers denote lower values than the lab reference (which are provided in parentheses). ACTH: adrenocorticotropic hormone; TSH: thyroid-stimulating hormone; FSH: follicle-stimulating hormone; min: minute; LH: luteinizing hormone; IGF-1: Insulin-like growth factor 1; PRL: Prolactin; PEG:  polyethylene glycol precipitation

Tests	Patient Value (Reference Value/Range)				
	April 30, 2024	May 1, 2024	May 2, 2024	May 3, 2024	June 26, 2024
8 am cortisol (nmol/liter)	63 (133-537)	-	-	-	-
Short synacthen test (nmol/liter)	-	0 min: 108, 30 min: 223, 60 min: 225 (>450)	-	-	-
ACTH (ng/liter)	24.8 (7.2-63.3)	-	-	-	-
Free T3 (pmol/liter)	-	-	-	2.0 (3.1-6.8)	1.6 (3.1-6.8)
Free T4 (pmol/liter)	-	-	-	9.4 (11.9-21.6)	10.3 (11.9-21.6)
TSH (mIU/liter)	2.380 (0.27-4.20)	-	-	1.780 (0.27-4.20)	0.877 (0.27-4.20)
FSH (U/liter)	-	-	< 1.0 (1.7-7.7 in luteal phase, 3.5-12.5in follicular phase)	-	< 1.0 (1.7-7.7 in luteal phase, 3.5-12.5 in follicular phase)
LH(U/liter)	-	-	< 1.0 (1-0-11.4 in luteal phase, 2.4-12.6 in follicular phase)	-	< 1.0 (1-0-11.4 in luteal phase, 2.4-12.6 in follicular phase)
IGF-1 (µg/liter)	-	-	< 25 (169-400 for tanner stage 5, female)	-	-
PRL (µg/liter)	-	-	785 (7.0-32.9)	682 (7.0-32.9)	-
PRL after PEG (µg/liter)	-	-	-	559.3 (3.5-28.6)	-

The patient was referred to ophthalmology and neurosurgery; Goldmann visual field test revealed right homonymous hemianopia while neurosurgical opinion posited a high risk of complications from surgical intervention, including brain biopsy (offered due to the location, aggressiveness, and radiological findings suggesting a differential diagnosis of a high-grade glioma). The family was thus advised that the best course of action was for the patient to undergo a repeat MRI brain in three months to monitor tumor size. The family agreed with such a conservative approach, and a brain biopsy was not performed.

**Figure 2:**
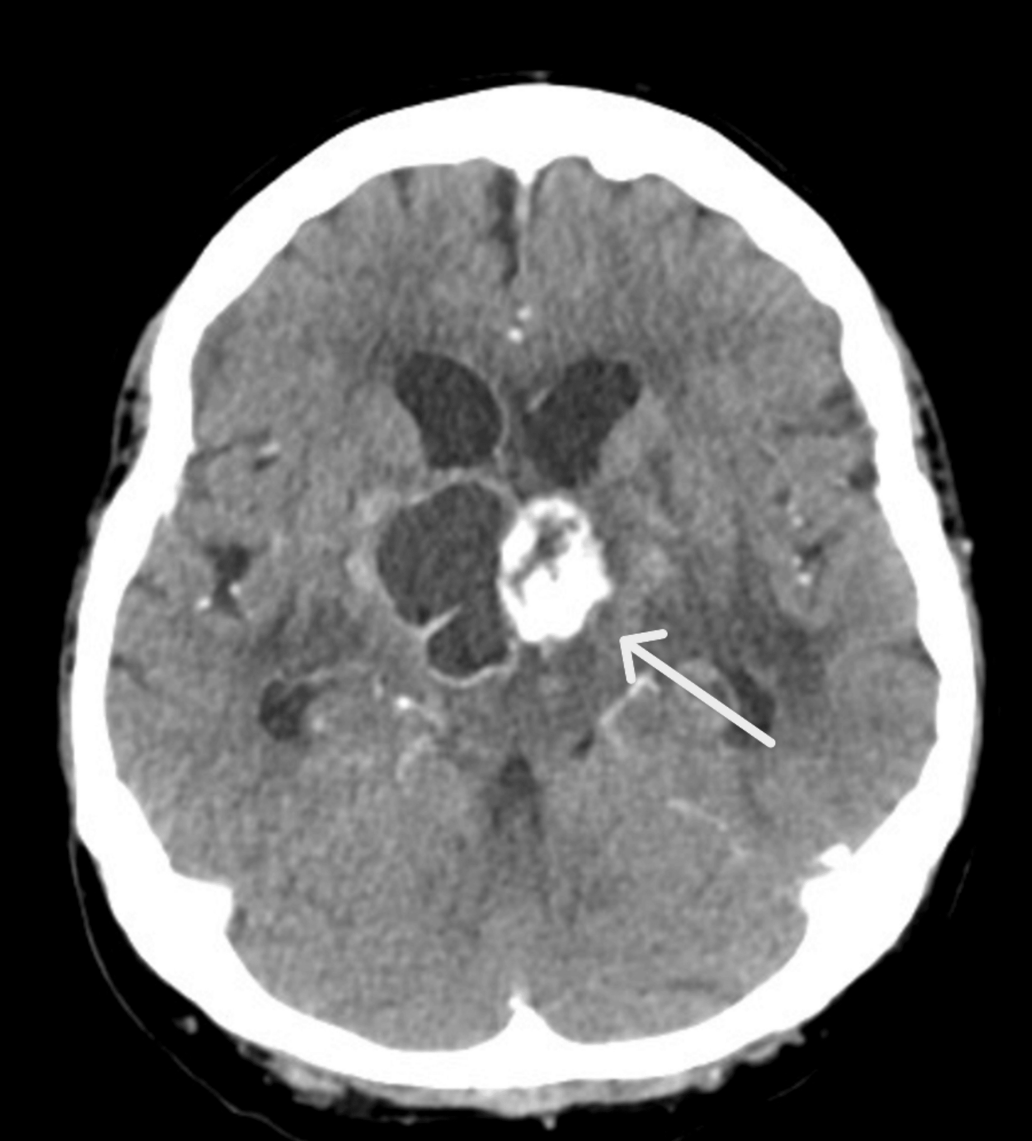
CT brain (axial view) with contrast performed on July 1, 2024 The known solid-cystic suprasellar-hypothalamic mass is larger in size, now measuring 3.8x3.5x2.9 cm. There is interval increase in the perilesional edema and local mass effect with compression of the floor of the third ventricle. New mild mass effect on the right frontal horn as well as interval increase in the size of the ventricles is noted, with prominence of bilateral temporal horns, suggesting early hydrocephalus. No acute intracranial hemorrhage or established territorial infarcts are noted.

A trial of therapy with the dopamine agonist cabergoline 0.25 mg twice a week was started on May 4, 2024, together with levothyroxine and hydrocortisone replacement. Although the location of the tumor was atypical for a prolactinoma, given the significant risks of neurosurgery, this was a reasonable initial course of action. She was discharged after a prolonged hospitalization but returned soon after at the end of June 2024 for worsening visual hallucinations and lethargy, together with low-grade fevers without localizing infective symptoms. She was treated for a *Klebsiella pneumoniae* lower urinary tract infection, and brain scans were repeated while inpatient. An eventual CT brain (Figure 2) showed continued enlargement of the suprasellar mass in a matter of just two months, this time, with the added complications of tumor bleed and early signs of hydrocephalus. The differential diagnosis, in view of an absence of response to cabergoline and rapid enlargement of the hypothalamic mass with sparing of the sella turcica, included a high-grade glioma or metastasis from prior renal cell carcinoma. 

In view of the progressive deterioration in mentation, neurological status, and function, as well as the patient’s history of multiple comorbidities, conservative management was most appropriate, with cessation of hormonal replacements and palliation of symptoms. Neurosurgical opinion remained that the risks of debulking tumor surgery or brain biopsy were prohibitive. Despite administration of intravenous dexamethasone and antiemetics for recurrent vomiting, minimal improvement was observed. Eventually, dialysis was withdrawn, and the patient was terminally discharged on July 5, 2024, with eventual demise four days later. 

## Discussion

For the internist who might occasionally see prolactinomas and non-prolactin secreting masses under his or her care, there are several learning points. With dopamine agonist therapy, normalization of prolactin levels and tumor shrinkage occur in the vast majority of patients with prolactin-secreting microadenomas and macroadenomas, with shrinkage of macroprolactinomas in 92% of patients within six months and normalization of prolactin levels in 80-85% of patients within two years [6,7]. When prolactin levels do not show significant decline with adherent dopamine agonist treatment, or when tumor size does not reduce sufficiently, alternative diagnoses should be entertained. One such differential diagnosis is of metastasis to the pituitary gland, which tends to occur in elderly patients with breast, lung, and other solid tumors, usually running an indolent course, and presenting with diabetes insipidus rather than compressive or focal neurological symptoms [8]. Aggressive pituitary tumors or carcinomas may invade surrounding tissues and lead to cerebrospinal or systemic metastases when not treated appropriately with radiotherapy or surgical resection [9].

Hyperprolactinemia in chronic kidney disease (CKD) without an underlying prolactinoma is fairly common, with worsening renal impairment correlating with higher prolactin levels, and several studies showing prolactin levels to be as high as around 100 µg/liter in ESRD patients [10]. Common mechanisms include reduced renal clearance, altered hypothalamic-pituitary axis from chronic uremia, downregulation of dopamine receptors, and medications. Although one case report found prolactin suppression of >90% with initial bromocriptine and later cabergoline treatment in an ESRD patient with severely elevated prolactin levels of 2056 µg/liter [11], there does not appear to be a consistent trend and assurance that dopamine agonism will always severely reduce hyperprolactinemia in CKD or ESRD patients without prolactinomas. 

In the setting of known malignancy or metastatic disease with new neurological symptoms, it is prudent to perform brain imaging to rule out intracranial causes, before referring to medical and surgical specialists to decipher and clinch an accurate diagnosis to initiate treatment. However, it is also worthwhile to eventually consider the goals of care to prevent misuse of limited resources, especially in developing countries. In our case, the patient unfortunately had developed metastatic disease from her prior renal cell carcinoma or was harboring a high grade glioma to explain most of her eventual symptoms.

## Conclusions

Hyperprolactinemia remains a red herring in ESRD patients, with higher levels often prevalent in patients that do not harbor prolactinomas. In such instances, alternative diagnoses, including malignancies and metastatic disease, should be entertained. Although it was unfortunate that our patient deteriorated rather rapidly after her initial diagnosis and treatment, we find solace in the fact that other physicians could learn from this predicament and apply these learning points to the next patient who presents with visual hallucinations and has a suprasellar mass.
